# Quantitative Analysis of Apisin, a Major Protein Unique to Royal Jelly

**DOI:** 10.1155/2016/5040528

**Published:** 2016-09-18

**Authors:** Takako Furusawa, Yasuko Arai, Kenji Kato, Kenji Ichihara

**Affiliations:** Nagaragawa Research Center, API Co., Ltd., 692-3 Nagara, Gifu 502-0071, Japan

## Abstract

Apisin, a protein that is unique to royal jelly (RJ), is known to compose the greater part of the RJ proteins and to exist as a heterooligomer containing major royal jelly protein 1 and apisimin. However, few reports on the methods for quantifying apisin have been published. Thus, we attempted to quantify apisin using HPLC, a widely used analytical technique, as described below. Isoelectric precipitation and size-exclusion chromatography were used to obtain the purified protein, which was identified as apisin by SDS-PAGE and LC-MS analyses. The purified apisin was lyophilized and then used to generate a calibration curve to quantify apisin in RJ. The apisin content was fairly constant (i.e., 3.93 to 4.67 w/w%) in natural RJ. This study is the first to describe a simple, standardized method for quantifying apisin using HPLC and suggests that apisin can be used as a benchmark for future evaluations of RJ quality.

## 1. Introduction

Queen honeybees have an approximately 2-fold larger body size and 10-fold longer lifespan than worker bees. Royal jelly (RJ) is a special food for queen bees, and royalactin, an RJ protein, has been reported to play an important role in the differentiation of queen bees [1]. In humans, RJ has been utilized in medicines, health-promoting foods, and cosmetics for many years. RJ primarily consists of water (60–70%), proteins (9–18%), lipids (3–8%), carbohydrates (7–18%), and other components [2]. Proteins, the second most abundant component of RJ, include the major royal jelly protein family (MRJP1 to MRJP9), apisimin, royalisin, and jelleins [3–7], among others, and MRJP1 is the major protein in RJ [8]. Royalactin is thought to be the same protein as MRJP1.

A 350 kDa glycoprotein from RJ exhibiting high-mannose-type N-glycosylation was first reported in 1995 [9]. In the next year, this glycoprotein was found to include two types of proteins with the following N-terminal sequences, as determined by N-terminal amino acid sequence analysis of its structural components: Asn-Ile-Leu-Arg-Gly- and Lys-Thr-Ser-Ile-Ser- [10]. This 350 kDa RJ glycoprotein was named “apisin” after* Apis mellifera *L. [11, 12]. Subsequently, another research group identified an oligomer consisting of MRJP1 and apisimin using sodium dodecyl sulfate-polyacrylamide gel electrophoresis (SDS-PAGE) and liquid chromatography-mass spectrometry (LC-MS) and suggested that the MRJP1 oligomer is the same as the 350 kDa RJ glycoprotein apisin [13]. In fact, the N-terminal Asn-Ile-Leu-Arg-Gly- and Lys-Thr-Ser-Ile-Ser- sequences, which were identified in the 350 kDa glycoprotein in 1996 [10], coincided with the N-terminal sequences of MRJP1 and apisimin, respectively. Thus, apisin is considered to be a unique, major protein complex in RJ that consists of MRJP1 and apisimin. Furthermore, recent research revealed that most MRJP1 exist as an oligomer in apisin [14]. Therefore, we predict that the apisin content can be used as an RJ quality standard. Several researchers have reported an enzyme-linked immunosorbent assay (ELISA) method for quantifying apisin [14–16]. However, the ELISA method directly measures only the amount of MRJP1, not apisin itself. In addition, few reports have addressed the direct quantification of apisin by high performance liquid chromatography (HPLC). One possible reason is the difficulty of preparing a pure, stable standard of apisin to construct a calibration curve for quantification. In previous reports, apisin was fractionated by anion-exchange gel chromatography [17] or ultracentrifugation [18] which is time consuming and requires a dedicated apparatus. Furthermore, the resulting apisin fraction was crude and produced multiple HPLC peaks.

In the present study, we attempted to establish a simple method for quantifying apisin by HPLC based on a standard curve. Then, we quantified the apisin content in various RJ products using the developed HPLC method.

## 2. Materials and Methods

### 2.1. RJ

Natural and lyophilized RJ, which originated from* Apis mellifera* L. in China, were obtained from API Co., Ltd. (Gifu, Japan), and stored at −20°C and 4°C, respectively, until further use. Fresh RJ was also collected from the Kawashima Apiary of API Co., Ltd. Briefly, female honeybee larvae (approximately 1 mm in length) were gently placed into individual artificial queen cells (diameter 10 mm × height 12 mm). After 72 h, the larvae were removed from the cells, and then fresh RJ was collected with a soft spoon. The fresh RJ was aliquoted into tubes containing 150 mg each and used immediately or stored at −20°C, 4°C, 25°C, and 40°C.

### 2.2. Isolation and Purification of Apisin

Natural RJ samples (3 g) were suspended in 100 mL of ultrapure water, and then the pH of the RJ suspension was increased from 4.0 to 6.0 by 0.2 by adding 10% (w/v) sodium hydroxide solution. Each pH-controlled suspension was centrifuged at 1,880 ×g for 20 min at 4°C to separate the supernatant and pellet. The supernatants were diluted to 100 mL with 0.1 M phosphate buffer containing 0.3 M NaCl (pH 7.2) by reference to a previous study [19], and the pellets were resuspended and diluted to 100 mL with the same solution. These samples were filtered and analyzed using size-exclusion columns (Protein KW-803, 8.0 mm I.D. × 300 mm; Protein KW-G, 6.0 mm I.D. × 50 mm, Showa Denko K.K., Tokyo, Japan) connected to an HPLC (Alliance, Waters, Massachusetts, USA) under the following conditions: a mobile phase of 0.1 M phosphate buffer containing 0.3 M NaCl (pH 7.2), an injection volume of 30 *μ*L, a flow rate of 0.3 mL/min, a column temperature of 30°C, and a wavelength of 280 nm. The column was calibrated using Gel Filtration Calibration Kit High Molecular Weight Proteins (GE Healthcare, Pittsburgh, USA). The degree (%) of purity of each sample was assessed by the ratio of the apisin peak area to the total peak area in the chromatograms.

The apisin-rich fraction was prepared by suspending the pellet in the buffer solution and was purified by size-exclusion column chromatography using Sephacryl S-300HR (25 mm I.D. × 460 mm, 225 mL, GE Healthcare, Tokyo, Japan) with a mobile phase of 0.1 M phosphate buffer containing 0.3 M NaCl (pH 7.2). The purity of the apisin fraction was further analyzed on an anion-exchange chromatography using a TSK gel DEAE-5PW column (TOSOH Corporation, Tokyo, Japan). The apisin fraction was collected, desalted by ultrafiltration with an Amicon Ultra-15 unit (NMWL 50,000, 15 mL, UFC910008, Merck Millipore, Darmstadt, Germany), added to ampoules with a 9 : 1 ratio of trehalose (D-(+)-trehalose anhydrous Tokyo Chemical Ind., Co., Ltd., Tokyo, Japan) to apisin, and then lyophilized. The net amount of apisin was calculated by subtracting the trehalose weight from the total weight, including the weight of the container.

In the stability test, the lyophilized apisin was preserved at −20°C or 4°C for 1, 3, 6, 9, and 12 months and was quantified using an internal standardization method at each point. The purified apisin and ovalbumin (Sigma-Aldrich Co., LLC., Missouri, USA), which was used as an internal standard, were dissolved in the mobile phase solution consisting of 0.1 M phosphate buffer and 0.3 M NaCl (pH 7.2) and analyzed by size-exclusion HPLC. The residual amounts of apisin are expressed as a percent of the initial (day 0) value.

### 2.3. Apisin Identification

The fractionated, purified protein was identified as apisin using SDS-PAGE, followed by LC-MS analysis. The test samples were dissolved in SDS sample buffer (SDS-SB; 62.5 mM Tris-HCl (pH 6.8) containing 10% (v/v) glycerol, 2% (w/v) SDS, and 50 mM dithiothreitol), vortexed, and then boiled for 3 min at 95°C. After measuring the protein concentrations using a bicinchoninic acid (BCA) protein assay kit (Pierce Biotechnology, Rockford, IL, USA) and adding the tracking dye bromophenol blue, 5 *μ*g of the protein was loaded onto 10–20% acrylamide SDS-PAGE gels (80 mm × 80 mm × 1 mm, BIO-CLAFT Co., Ltd., Tokyo, Japan) and electrophoresed with a constant current of 25 mA/gel for 70 min. A buffer consisting of 0.025 M Tris, 0.192 M glycine, and 0.2% (w/v) SDS was used as the running buffer. The proteins were visualized with Oriole Fluorescent Gel Stain (Bio-Rad Laboratories Inc., Hercules, CA, USA). The gel was calibrated by measuring the migration distances for standard proteins of known molecular masses (Precision Plus Protein*™* Dual Xtra Standards [Bio-Rad; Hercules, CA, USA]).

Each protein band on the gel was excised, washed with 100 mM ammonium bicarbonate (NH_4_HCO_3_) in 50% acetonitrile (ACN), dehydrated with 100% ACN, reduced with 50 *μ*L of 10 mM dithiothreitol/100 mM NH_4_HCO_3_ at 56°C for 30 min, and then alkylated with 50 *μ*L of 55 mM iodoacetamide/100 mM NH_4_HCO_3_ for 45 min at room temperature in the dark. The gel slices were then washed with 100 mM NH_4_HCO_3_ and dehydrated with 100% ACN, and the proteins were digested with 50 *μ*L of 0.01 g/L lysyl endopeptidase/100 mM NH_4_HCO_3_ (Lys-C, #125-05061, Wako Pure Chemical Industries, Ltd., Osaka, Japan) or with 50 *μ*L of 0.005 g/L aspartic protease/100 mM NH_4_HCO_3_ (Asp-N, #V162A, Promega K.K., Tokyo, Japan) at 37°C for 16 h (overnight). The peptides were extracted by sonication for 3 min at ambient temperature, followed by the addition of the extraction buffer (50% ACN containing 0.5% formic acid), and vortexed twice for 10 min each. The extracted peptides were dried in a speed vacuum, dissolved in 20 *μ*L of 0.1% formic acid by sonication, and then subjected to LC-MS analysis.

The peptides were separated on a Protein Separation Technology BEH300 C18 column (1.7 *μ*m I.D. × 150 mm, Waters Corporation, MA, USA) by binary gradient elution (from 0.1% formic acid/water to 0.1% formic acid/ACN in 80 min) at a flow rate of 0.2 mL/min via an ultraperformance liquid chromatograph (UPLC) connected to a liquid chromatograph-electrospray ionization quadrupole/time-of-flight mass spectrometer (LC-ESI-QTof MS) (ACQUITY UPLC coupled to Xevo G2 QTof, Waters). The LC-MS was operated in MS^E^ (positive/sensitivity) mode. The MS^E^ spectra were searched against the NCBI Taxonomy database, in which 64,296 proteins were registered with “Apis” (ID: 7459), using the ProteinLynx Global SERVER software (version 5.2, Waters). The parameters used in this analysis were as follows: (1) Lys-C or Asp-N as the protease, (2) one missed cleavage site, (3) carbamidomethylation of cysteine as a fixed modification, and (4) oxidation of methionine and tryptophan and phosphorylation of serine, threonine, and tyrosine as variable modifications. To identify the protein, at least 3 different theoretical y or b ions were assigned to one unique peptide, and a total of 7 different unambiguous y or b ions were necessary for the successful manual verification of the protein. The MS and MS^E^ ion intensity thresholds were 150 counts and 50 counts, respectively.

The protein bands on the SDS-PAGE gel were extracted using the alkaline extraction method [20]. Briefly, the gel was excised, washed with ultrapure water, and minced in 100 *μ*L of 100 mM NH_4_HCO_3_/50% ACN. After removing the NH_4_HCO_3_ solution, the gel was soaked in 50 *μ*L of 0.1 M NaOH for 10 min at 25°C to extract the protein and then was subjected to SDS-PAGE analysis or mass analysis of the intact protein. The intact protein was analyzed using LC-ESI-QTof MS as described below. The protein solution was diluted 2-fold with 0.1% formic acid, filtered, and separated on a Protein BEH C4 column (300 Å, 1.7 *μ*m I.D. × 100 mm, Waters) using a gradient elution with a water/ACN mobile phase (80 : 20 to 80 : 20 in 10 min) containing 0.1% formic acid at a flow rate of 0.2 mL/min. The MS results were analyzed using MAXENT I software (Waters).

### 2.4. Quantitation of Apisin in RJ

Natural RJ (150 mg) or lyophilized RJ (50 mg) was dissolved in 50 mL of 0.1 M phosphate buffer containing 0.3 M NaCl at pH 7.2 and centrifuged at 12,500 ×g for 5 min at 4°C. The supernatant was filtered (0.2 *μ*m), and apisin was quantified by size-exclusion HPLC analysis according to the methods described in Section 2.2, except for the wavelength 210 nm at which the peak intensity of apisin was higher, using a standard curve generated with known concentrations of the lyophilized apisin as the standard reference material. The experiments were performed in quadruplicate.

### 2.5. Stability of Apisin in RJ

As described above, fresh RJ was prepared from our bee (*Apis mellifera* L.) keeping farm. The fresh RJ was immediately used to test the stability of apisin at −20°C, 4°C, 25°C, and 40°C. The residual quantity of apisin at each temperature was measured by the standard curve method using size-exclusion HPLC after 1, 4, 7, 14, and 32 days. The experiments were performed in triplicate.

## 3. Results

### 3.1. Isolation and Purification of Apisin

**Figure 1:**
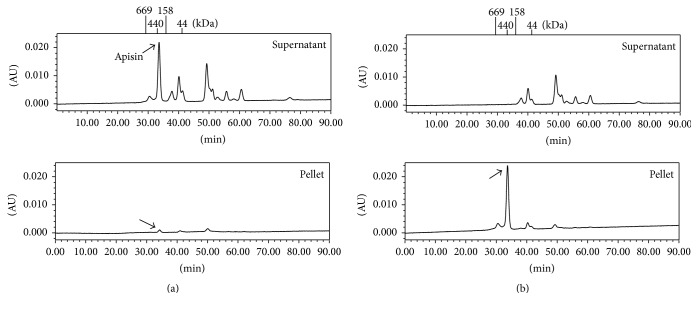
Fractionation of an apisin-rich protein by isoelectric precipitation. Elution profiles of apisin in the supernatant and pellet of the RJ solution at pH 3.9 (a) and pH 4.6 to 4.8 (b) using size-exclusion gel chromatography. The methodological details are provided in Section 2. The changes in the purity (%) of apisin in the supernatant and pellet at pH 3.9 to 6.0 are shown in Table 1.

The natural RJ was suspended in ultrapure water, and then the pH of the RJ suspension was increased from 4.0 to 6.0 by adding sodium hydroxide solution. As shown in Figure 1, the peaks indicated with arrows were attributed to apisin. At the natural pH of 3.9 (Figure 1(a)), apisin was detected in the supernatant fluids, whereas it was detected in the pellets at pH 4.8 (Figure 1(b)). The apisin-rich (>60%) protein fraction was obtained at pH values from 4.4 to 5.4 (Table 1).

**Figure 2:**
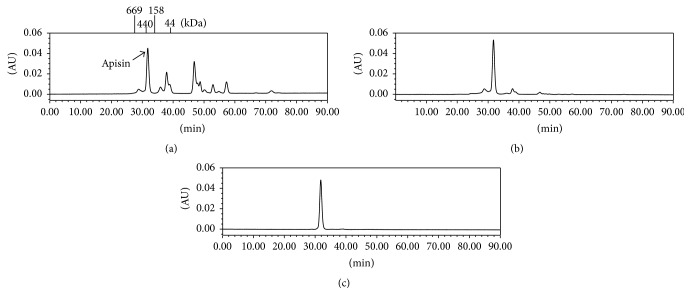
Purification of apisin by size-exclusion HPLC. Elution profiles of the RJ proteins obtained by size-exclusion gel chromatography: (a) natural RJ proteins, (b) proteins after isoelectric precipitation at pH 4.8, and (c) proteins after purification by size-exclusion HPLC.

The apisin-rich fraction, which was obtained by isoelectric precipitation at pH 4.8, was purified by size-exclusion column chromatography, and a single peak for apisin was observed in the HPLC chromatogram (Figure 2). Additionally, apisin was detected as a single peak on anion-exchange chromatography (data not shown).

**Figure 3:**
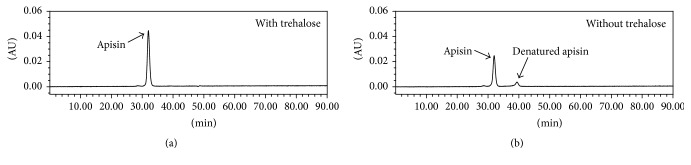
Elution profiles of apisin lyophilized with trehalose (a) and without trehalose (b) by size-exclusion gel chromatography using HPLC.

**Figure 4:**
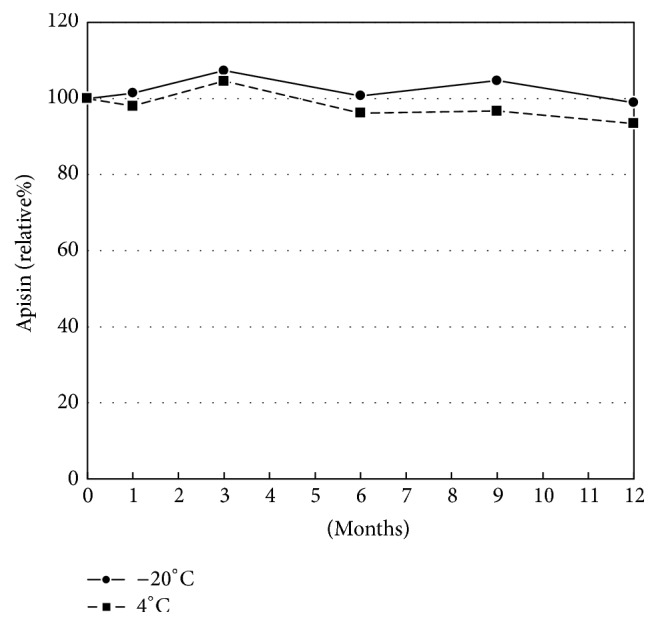
The stability of the lyophilized apisin powder. The lyophilized apisin was preserved at −20°C or 4°C for 1, 3, 6, 9, and 12 months, and the residual amounts of apisin are expressed as percentages of the initial (day 0) value. The methodological details are provided in Section 2.

**Figure 5:**
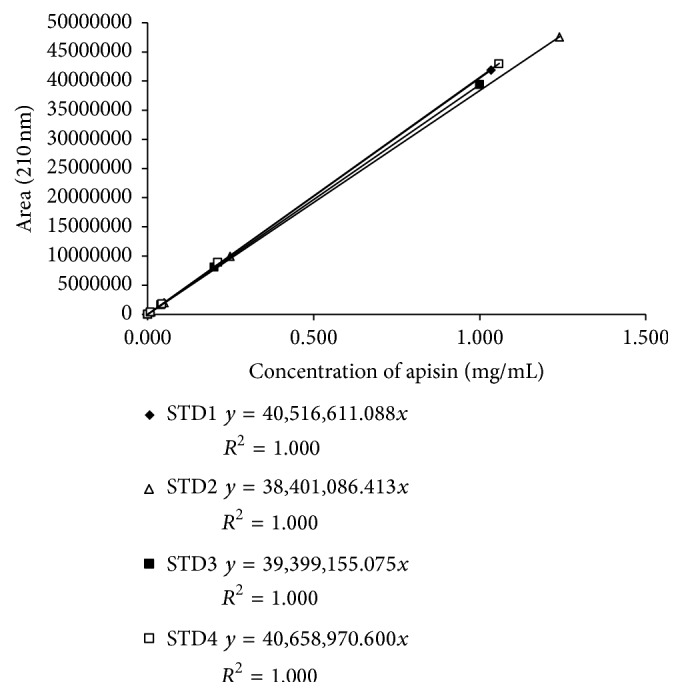
Calibration curves were determined using the lyophilized apisin powder. All of the calibration curves were linear (*R* ^2^ = 1.000) from 0.01 to 1.00 mg/mL. The experiments were performed in quadruplicate.

The purified apisin fraction was desalted and lyophilized with trehalose, which almost completely protected the apisin from lyophilization-induced denaturation (Figure 3). The lyophilized apisin was stable for at least 12 months when stored at 4°C or −20°C (Figure 4). The calibration curve constructed from the lyophilized apisin showed good linearity (Figure 5). These findings suggested that lyophilized apisin could be used as a standard reference material for quantification.

### 3.2. Identification of the Purified Protein as Apisin

**Figure 6:**
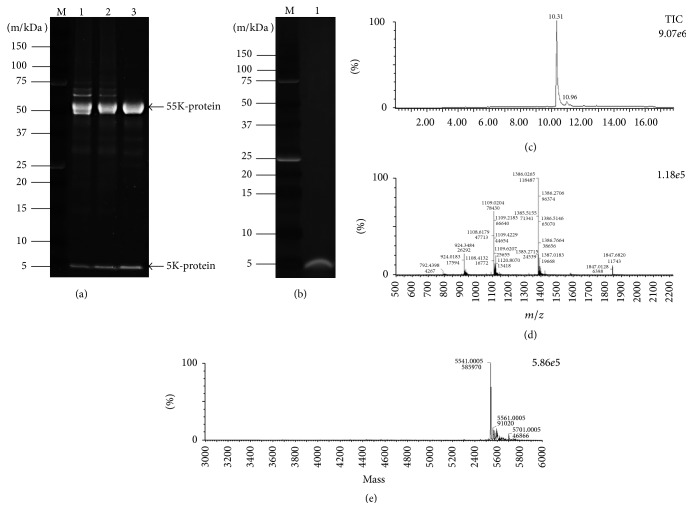
Identification of apisin by SDS-PAGE and LC-MS. (a) SDS-PAGE profiles of natural RJ (lane 1), the isoelectric point-precipitated protein (pH 4.8) (lane 2), and the protein purified by size-exclusion HPLC (lane 3). (b) SDS-PAGE profile of the protein extracted from the 5 kDa band (lane 1). (c) Total ion chromatogram of the 5 kDa protein. (d) Mass spectra of the 5 kDa protein. (e) Calculated molecular weight of the 5 kDa protein according to MAXENT I analysis. The methodological details are provided in Section 2.

**Figure 7:**
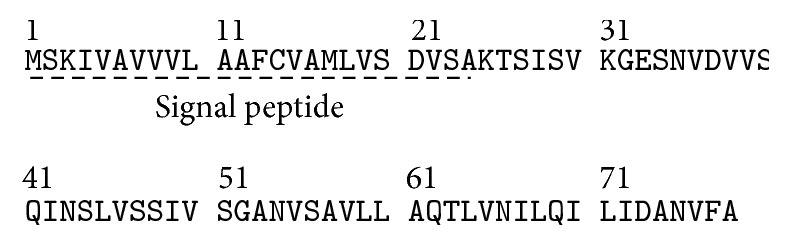
Amino acid sequence of apisimin [*Apis mellifera*, NP_001011582.1].

SDS-PAGE and LC-MS analyses were performed to identify the purified protein as apisin. The SDS-PAGE results for natural RJ, the isoelectric point (4.8) precipitated proteins, and the purified proteins are shown in Figure 6(a). The purified protein (lane 3) migrated as approximately 55 kDa and 5 kDa species on SDS-PAGE gels. The 55 kDa protein was identified as MRJP1 by a proteomics approach involving Lys-C treatment. However, the 5 kDa protein could not be identified by this approach and was instead subjected to de novo sequencing. The 5 kDa protein was digested with Lys-C or Asp-N, and the following three peptides were detected: “KTSISV or KTSLSV,” “KTSISVKGESNVD or KTSLSVKGESNVD,” and “DANVFA” (Table 2). After searching these sequences against the Taxonomy NCBI database, only “KTSISV,” “KTSISVKGESNVD,” and “DANVFA” shared an identical amino acid sequence with apisimin, which has a theoretical mass of 5,540 Da (all the amino acid sequences of mature apisimin are shown in Figure 7). The KTSLSV peptide was assigned to A0A088AFZ8_APIME uncharacterized protein, with a theoretical mass of 26,380 Da; KTSLSVKGESNVD did not match any homologous protein families. Next, the 5 kDa protein was extracted from the SDS-PAGE gel and used for intact protein mass analysis (Figure 6). The accurate mass of the 5 kDa protein was estimated to be 5,540.0 Da (Figure 6(e)). The theoretical mass of apisimin is believed to be 5,540 Da, and it has been reported to have a mass of 5,540.4 or 5,540.9 [5, 13]. Taken together, these findings confirm that the purified protein is a two-protein complex consisting of MRJP1 and apisimin.

### 3.3. Quantitation of Apisin in RJ

We demonstrated that apisin could be accurately quantified with a standard curve using HPLC. The apisin content in natural and lyophilized RJ was 3.93 to 4.67% (w/w) and 10.93 to 11.86% (w/w), respectively (Table 3), indicating that the apisin content is fairly constant in RJ. Incidentally, the water content in natural and lyophilized RJ was 63.5 to 65.8% (w/w) and 1.4 to 2.4% (w/w), respectively, when measured by a conventional vacuum-drying method (70°C for 4 h).

### 3.4. Stability of Apisin in RJ

**Figure 8:**
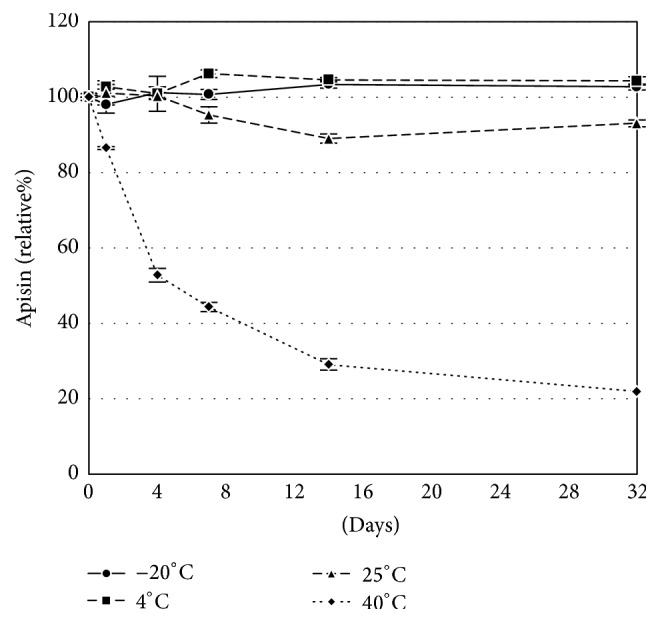
The stability of apisin in RJ. Fresh RJ was preserved at −20°C, 4°C, 25°C, or 40°C for 1, 4, 7, 14, and 32 days, and the residual amounts of apisin were quantified with a standard curve using size-exclusion HPLC and expressed as percentages of the initial (day 0) value. The methodological details are provided in Section 2. All measurements were performed in triplicate, and the data represent the mean ± S.D.

We assessed the residual quantity of apisin in fresh RJ after preservation at various temperatures. The quantity of apisin was unchanged at −20°C and 4°C for at least 32 days but decreased by approximately 10% and 80% at 25°C and 40°C, respectively (Figure 8).

## 4. Discussion

Here, we successfully demonstrated the first HPLC-based method for the accurate quantitation of the RJ protein apisin. As shown in Figure 1, we identified the chromatographic peak indicated by an arrow as apisin because apisin exhibited the highest peak on supercritical fluid chromatography at 280 nm, and its molecular weight was estimated to be 280 to 420 kDa in previous studies [9, 13, 21, 22].

Initially, we attempted to fractionate crude apisin by isoelectric precipitation at the optimum pH values. Apisin is a heterooligomer containing MRJP1 and apisimin, and their theoretical isoelectric points were computed to be 5.03 and 4.55, respectively, using the ExPASy Compute pI/Mw tool (http://web.expasy.org/compute_pi/) based on their amino acid sequences. Based on these isoelectric points, the RJ proteins were precipitated at pH values ranging from 4.0 to 6.0, whereas the apisin-rich protein fractions were obtained at pH values from 4.4 to 5.2; thus, as expected, apisin has an isoelectric point of approximately 4.6. The apisin-rich fraction was also purified by size-exclusion gel chromatography, which is frequently used to separate various biomaterials, such as proteins and polysaccharides, according to their molecular sizes. Ultimately, we were able to derive apisin, which was detected as a single peak in the HPLC chromatogram, from the RJ proteins. To the best of our knowledge, this is the first report of the isolation of apisin using isoelectric precipitation followed by size-exclusion gel chromatography. Isoelectric precipitation is a commonly used, rapid, and simple method for fractionating individual proteins. In addition, this relatively easy method provides a large amount of the apisin-enriched fraction, which could be used for additional functional, physicochemical, and analytical studies of RJ.

We then identified the purified protein as apisin using SDS-PAGE and LC-MS analyses. The protein migrated as approximately 55 and 5 kDa species on SDS-PAGE gels. Using a proteomics approach and automatic software-based analysis, the 55 kDa protein was identified as MRJP1, but we failed to identify the 5 kDa band as apisimin. The reasons may include the following: (1) the sequences of the mature protein and the protein in the database, which includes a signal peptide sequence, differ. (2) Only one or two detectable peptides can be generated by the LC-MS analysis because two cleavage sites—Lys-C and Asp-N—exist in the apisimin sequence. (3) The detectable peptides include Ile, which can be distinguished from Leu by LC-MS analysis (Table 2). Thus, we ultimately identified the 5 kDa protein as apisimin via de novo analysis and intact protein mass measurements. Therefore, we determined that the purified protein is indeed apisin, a two-protein complex consisting of MRJP1 and apisimin. Interestingly, the MRJP1 oligomer has been reported to consist of the 55 kDa protein MRJP1 and the 5 kDa protein apisimin [13], suggesting that our purified protein is the same protein as the MRJP1 oligomer, which is also called apisin (350KRJGP).

Third, we tried to measure the apisin content in various RJ products. The apisin fraction that was purified by gel chromatography was desalted and lyophilized with trehalose to obtain a dried apisin powder, which was used as a standard reference material. Trehalose is typically used to prevent the lyophilization-induced denaturation of proteins [23]. Indeed, trehalose prevented the denaturation of apisin (Figure 3). The lyophilized apisin powder was stable for at least 12 months at 4°C or −20°C and allowed us to generate a linear calibration curve for the HPLC analysis, suggesting that the prepared apisin powder can be used as a reference standard for the quantitative analysis of apisin by HPLC. Here, we found that the apisin content in RJ was fairly constant, in the ranges of 3.93 to 4.67% (w/w) for natural RJ and 10.93 to 11.86% (w/w) for lyophilized RJ. We also found that the apisin content in RJ did not change for at least 32 days when it was stored at −20°C, 4°C, or 25°C but decreased by approximately 80% at 40°C after 32 days. Previous studies revealed that RJ lost the ability to enhance the proliferation of rat hepatocytes when stored at 40°C for 7 days and suggested that the thermal denaturation of royalactin, which is assumed to be the same protein as MRJP1, is responsible for this phenomenon [1, 19].

Currently, the content of 10-hydroxy-2-decenoic acid (10-HDA), which is a chemically stable fatty acid unique to RJ, is required for the specification of RJ products. However, a potential disadvantage of using 10-HDA is that the amount of 10-HDA in RJ decreases during the production process and can be manipulated by adding an extraneous 10-HDA-rich RJ fraction. In contrast, the content of apisin was fairly constant among RJ products, for example, the fresh RJ from our bee farm, the imported RJ from China, and the lyophilized RJ. Thus, there is a possibility that the MRJP1 oligomer apisin can be used as a benchmark for future evaluations of RJ quality in addition to 10-HDA, although there are a few critical issues that must be overcome relating to achieving a secure supply of the apisin reference standard for quantitative analysis.

In conclusion, we developed the first method for the rapid isolation and purification of the RJ protein apisin and then quantified this protein by HPLC using a standard curve. This analysis revealed that the apisin content is fairly constant among natural RJ samples.

## Figures and Tables

**Table 1:** Changes in the apisin purity (%) in RJ suspensions at various pH values.

pH	3.9	4.0	4.2	4.4	4.6	4.8	5.0	5.2	5.4	5.6	5.8	6.0

Supernatant	27.6	27.7	27.9	16.9	0.0	0.0	0.7	2.1	13.0	24.6	27.0	26.6

Pellet	28.8	33.6	49.2	66.0	70.3	66.5	67.7	67.4	60.5	32.2	44.5	15.1

**Table 2:** List of identified peptides corresponding to the 5 kDa protein.

Treated enzyme	Peak R.T. (min)	Sequences	Matched proteins
Lys-C	11.04	TSISVK	Apisimin (5,540 Da)
		TSLSVK	A0A088AFZ8_APIME Uncharacterized protein (26,380 Da)

Asp-N	12.60	KTSISVKGSNV	Apisimin (5,540 Da)
		KTSLSVKGESNV	Not applicable

Asp-N	15.25	DANVFA	Apisimin (5,540 Da)

These sequences were searched against the NCBI Taxonomy database (the classification “Apis”, ID: 7459).

**Table 3:** The apisin content in natural and lyophilized RJ.

Samples	Lot number	Apisin (%)
Natural RJ	I	4.62 ± 0.11
	II	4.43 ± 0.10
	III	4.67 ± 0.11
	IV	4.39 ± 0.10
	V	3.93 ± 0.09
	VI	4.41 ± 0.10
	VII	4.62 ± 0.11

Lyophilized RJ	A	11.63 ± 0.29
	B	11.27 ± 0.27
	C	11.39 ± 0.27
	D	10.93 ± 0.24
	E	11.03 ± 0.24
	F	11.86 ± 0.28

Apisin was quantitated using a standard curve generated with known concentrations of lyophilized apisin. The methodological details are provided in Section 2. All measurements were performed in triplicate, and the data represent the mean ± S.D.
